# Thymoma-Associated Pleural Effusion Treated With Neoadjuvant Chemotherapy

**DOI:** 10.7759/cureus.63463

**Published:** 2024-06-29

**Authors:** Sreshta Paranji, Aatif Khurshid, Pritika Sharma, Rathnamitreyee Vegunta, Michael Fanucchi

**Affiliations:** 1 Internal Medicine, Westchester Medical Center, Valhalla, USA; 2 Hematology/Oncology, Westchester Medical Center, Valhalla, USA

**Keywords:** neoadjuvant chemotherapy (nact), masaoka staging, paraneoplastic syndromes, anterior mediastinal mass, malignant thymoma

## Abstract

Anterior mediastinal masses, including thymomas, can present with thoracic symptoms or paraneoplastic syndromes, especially in adults over 40. Diagnosis involves imaging and biopsy, and treatment includes surgical resection and chemotherapy, depending on the stage. A 31-year-old male, with a history of alcohol use disorder and a former smoker, presented with increasing heartburn, shortness of breath, left shoulder pain, and chest pain. Imaging revealed an anterior mediastinal mass with pleural thickening and a small effusion. A biopsy confirmed a B2-type thymoma. Initial treatment included cyclophosphamide, doxorubicin, and cisplatin, resulting in significant tumor reduction and pleural effusion resolution. The patient underwent planned surgical resection following neoadjuvant chemotherapy. This case highlights the complexity of advanced thymoma treatment and the effectiveness of neoadjuvant chemotherapy in reducing tumor burden, the associated effusions, and improving outcomes. Continuous follow-up and further studies are essential to optimize treatment protocols for advanced thymoma.

## Introduction

Mediastinal masses can range from benign to malignant, and, when faced with an anterior mediastinal mass, the differential can be wide. However, the most common culprits of anterior mediastinal mass include lymphomas, teratomas, thyroid, or thymomas [1]. Of these, thymomas are the most common in the adult population (women and men over 40 years old), as they account for 20-30% of anterior mediastinal masses in this demographic. Regardless, it only accounts for less than one percent of all malignancies in these patients [2]. There is a similar incidence between males and females. 

Clinically, 60% of patients with an anterior mediastinal mass are symptomatic on presentation [3]. The presentation of thymomas is generally either with thoracic manifestations or with paraneoplastic syndromes. Regarding thoracic manifestations, 60% of patients present with cough [4]. Other symptoms include dyspnea, voice hoarseness, and dysphagia [5]. More bulky mediastinal tumors may cause compression of the superior vena cava (SVC), leading to SVC syndrome including periorbital and facial edema, and upper extremity swelling; however, this is a rare case with thymomas [5]. Paraneoplastic syndromes associated with thymoma include myasthenia gravis, pure red cell aplasia, vasculitides, and hypogammaglobulinemia [1], with myasthenia gravis being the most common presenting paraneoplastic syndrome (30-50%) [4]. Myasthenia gravis is an autoimmune disorder caused by acetylcholine receptor antibodies notably associated with less aggressive thymomas. Other prognostic factors include resection status, tumor size (with size greater than 10 centimeters being a negative prognostic factor), and age at presentation (with age younger than 30 years being a negative prognostic factor) [6]. The WHO (World Health Organization) histologic staging has predictive value in prognosis as well; this staging ranges from A to C. The histologic description of A is medullary thymoma and AB is mixed thymoma, both of which have a 100% survival rate at 10-year follow-up [7]. B1 is predominantly cortical thymoma and B2 is cortical thymoma, both of which have an 83% survival rate at 10 years [7]. Those with the worst prognosis are B3, well-differentiated thymic carcinoma, and C, thymic carcinoma. The 10-year survival rates for these are 35% and 28%, respectively [7]. 

Initial diagnostic evaluation should involve thoracic imaging with a CT scan and/or MRI, with MRI being superior to CT because of its ability to distinguish between cystic and solid lesions [1]. Ultimately, the definitive diagnosis is made with a tissue biopsy. If a patient is thought to have a resectable mass, surgical resection should be done which can serve as both a diagnosis and initial step in management.

## Case presentation

**Figure 1:**
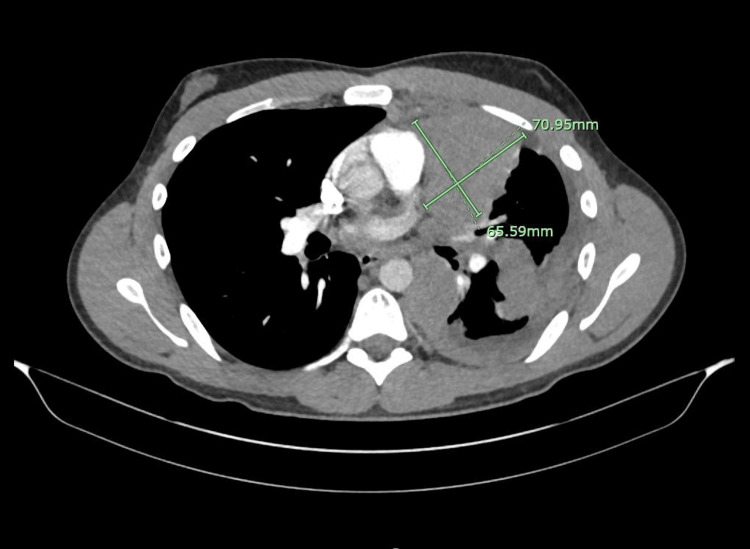
An axial cut demarcating the anterior mediastinal mass with measurements.

**Figure 2:**
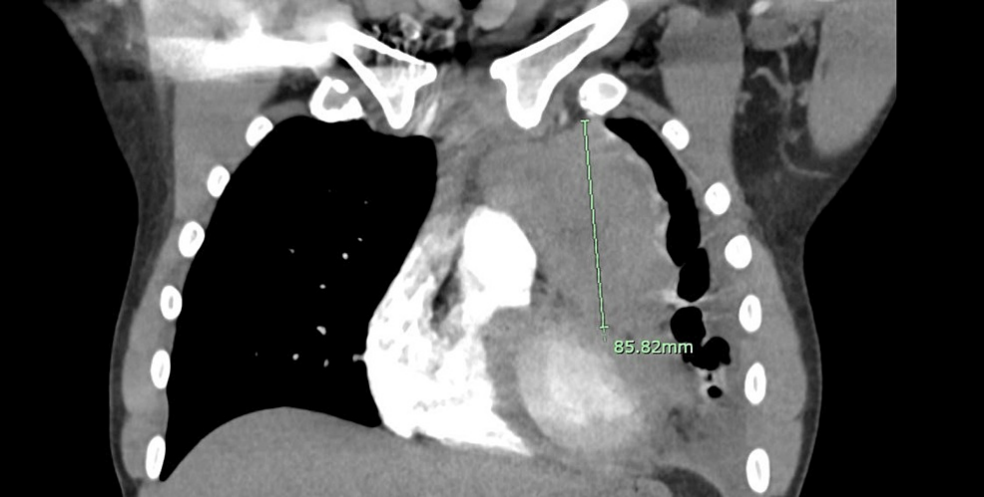
A coronal cut demarcating the mass.

A 31-year-old man with a past medical history of alcohol use disorder and a former smoker (quit one year ago, less than a one-pack year history) presented with increasing severity of heartburn associated with shortness of breath, left shoulder pain, and left-sided chest pain for one week. He had tried over-the-counter medications such as Tylenol with no improvement in the pain. He previously presented one month before another hospital with milder but similar symptoms and had been referred to a gastroenterologist at that time. His physical exam was unremarkable, including his vital signs and lung auscultation. His labs were significant for a lactate dehydrogenase (LDH) of 585 U/L (normal: 125-220 U/L) with a notably normal alpha-fetoprotein (AFP) level. A CT scan of his thorax revealed an anterior mediastinal mass with extensive pleural-based soft tissue thickening in the left lung extending into the fissure (Figures 1-2) and an adjacent loculated small left pleural effusion.

**Figure 3:**
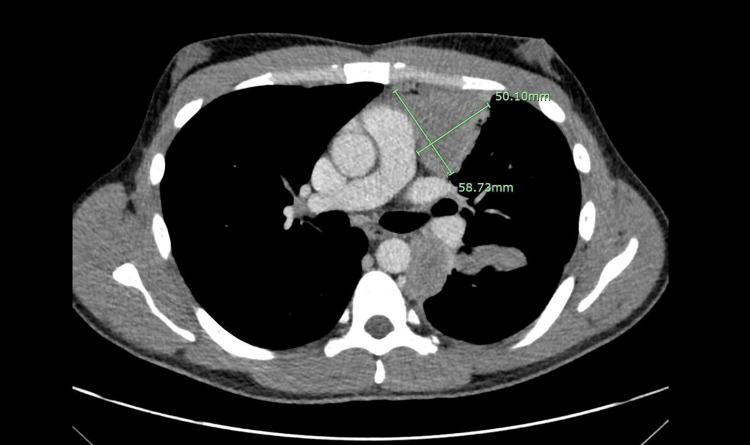
An axial cut after two cycles of chemotherapy demarcating the mass with measurements showing a reduction in tumor burden.

**Figure 4:**
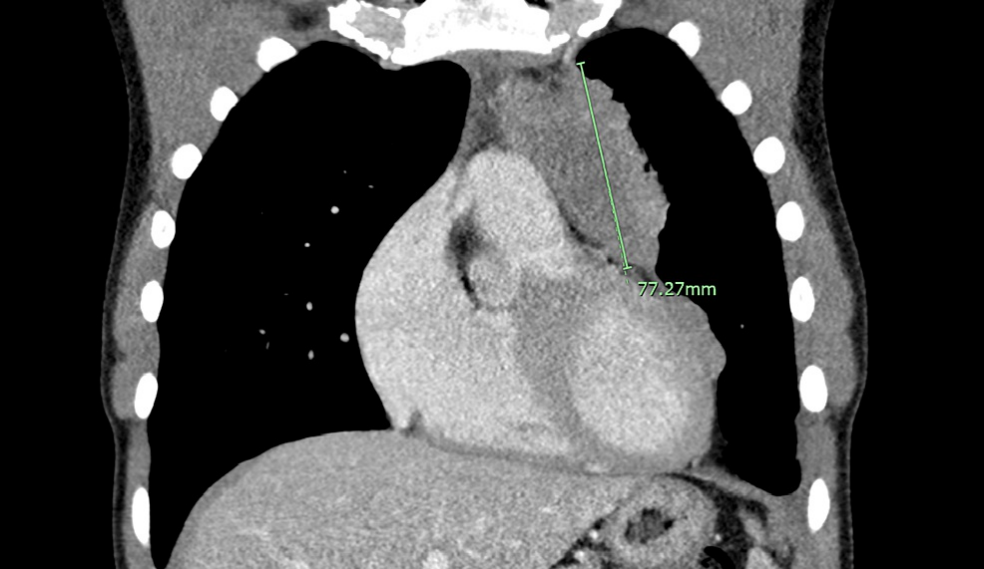
A coronal cut demarcating the mass after two cycles of chemotherapy.

A CT scan of his abdomen and pelvis done to evaluate the spread of the disease did not note any lymphadenopathy or additional masses. He underwent interventional radiology-directed biopsy of his anterior mediastinal mass. The pathology showed predominantly CD3+ T-lymphocytes with the majority expressing terminal deoxynucleotidyl transferase (TdT) and CD1a with CD4 and CD8 dual expression. There were also pan-cytokeratin highlights revealing clusters of epithelial cells, all consistent with a thymoma, B2 type. His cell-free DNA was negative for microsatellite instability-high (MSI-H) with no targetable mutations. Given pleural effusions, his staging was Stage IV according to the modified Masaoka and/or Stage IVa according to the American Joint Committee on Cancer (AJCC) tumor size, nodal spread, and metastatic spread (TNM) staging. The patient was clinically stable for discharge with an LDH of 314 U/L and was planned to receive neoadjuvant chemotherapy outpatient. Within one week of discharge, he was started on cyclophosphamide, doxorubicin, and cisplatin. After completing two cycles, a repeat CT scan demonstrated partial remission (PR) with a 44% decrease in the size of his anterior mediastinal mass (Figures 3-4).

Another 12% reduction in the tumor size was achieved after two more cycles. There was also improvement in the left pleural disease and interval resolution of the pleural effusion. The patient received five cycles of chemotherapy before his planned resection, with a plan for him to follow up outpatient for consideration of adjuvant chemotherapy with or without radiation afterward.

## Discussion

As in the case of most tumors and/or masses, the management recommendations regarding thymomas are based on the staging. Given the rarity of the disease, these recommendations cannot be based on clinical trials. The Masaoka staging ranges from stage I, encapsulated, to stage IVB, lymphatic or hematogenous metastasis. In patients with early-stage (Masaoka stage I or II) disease, surgical resection is the treatment of choice along with neoadjuvant chemotherapy [4]. The typical chemotherapeutic agents used are cyclophosphamide, doxorubicin, and cisplatin, which were given to our patient. After surgery, consolidation chemoradiation (chemoRT) is typically offered, and these patients have a five-year survival rate of 95% [5].

In the case of locally invasive or metastatic disease (Masaoka staging III-IVb), chemotherapy and radiation are the initial options of choice [4]. The chemotherapy regimen for this usually consists of cisplatin, vincristine, doxorubicin, and etoposide because of more advanced disease [8]. The Masaoka stages IVa (pleural or pericardial dissemination) and IVb (distant metastases) carry a worse prognosis, with a five-year-survival rate ranging from 11% to 50% [4], and the data for patients in these categories is varied when it comes to surgical resection. Typically, response to chemotherapy is tested following several cycles of neoadjuvant chemotherapy, and if the disease has responded enough to be considered resectable, surgical intervention is performed +/- postoperative chemotherapy or radiation therapy [9]. Even in patients with unresectable advanced disease, there seems to be a mortality benefit in patients who are appropriate surgical candidates to debulk their tumor, along with chemoRT [10]. 

It is important to discuss the outcomes of associated paraneoplastic syndromes after a thymectomy. While thymectomies can theoretically lead to the resolution of paraneoplastic syndromes, in practice, this may not be the case. For patients with a thymoma and myasthenia gravis, thymectomy typically lessens the severity of the myasthenia gravis with complete or pharmacologic remission in almost all patients; however, some symptoms persist in most patients [11]. Similarly, in patients with thymoma-associated pure red cell aplasia, resolution of anemia with only surgical excision of the thymoma without concomitant long-term medication therapy is rare [12].

In our patient, who presented without evidence of paraneoplastic syndromes, his staging was Masaoka stage IVa given the evidence of adjacent pleural effusion. The pleural effusion was eventually thought to be due to the thymoma after ruling out other causes such as heart failure, and so on, based on medical history, physical exam, and laboratory findings. This specific presentation of pleural effusion with thymoma makes our case unique, as it has only been seen a handful of times in the literature. The Masaoka staging dictated the neoadjuvant treatment, and the patient had a good response with not only a post-chemotherapy reduction in the mediastinal mass but also the complete resolution of the pleural effusion. These results are reassuring ahead of the resection, as normally late Masaoka staging has been significantly associated with incomplete resection [13]. He received five cycles of neoadjuvant chemotherapy before the planned resection. He will potentially be offered postoperative chemotherapy and radiation afterward to maintain sustained remission.

## Conclusions

Thymomas often present with localizing symptoms and/or paraneoplastic syndromes. This patient, who presented with one localizing symptom, his left shoulder pain, is a reminder to keep a high index of suspicion despite uncommon presenting symptoms and findings, such as his pleural effusion. As this patient’s course is postoperatively followed, it can be acknowledged that further studies are necessary to streamline management in patients with thymoma, especially advanced disease. We remain optimistic about this patient’s care given his response to neoadjuvant chemotherapy and hope that this case can add to the available evidence for the management of advanced-stage thymoma that includes preoperative medical treatment, surgical resection, and postoperative follow-up.
